# Case report: A novel *COL3A1* variant in a Colombian patient with isolated cerebrovascular involvement in vascular Ehlers–Danlos syndrome

**DOI:** 10.3389/fmed.2024.1304168

**Published:** 2024-03-26

**Authors:** Valeria Valencia-Cifuentes, Stiven Ernesto Sinisterra-Díaz, Valentina Quintana-Peña, Edgar Folleco, José A. Nastasi-Catanese, Harry Pachajoa, Juan P. Fernández-Cubillos

**Affiliations:** ^1^Department of Neurology, Fundación Valle del Lili, Cali, Colombia; ^2^Facultad de Ciencias de la Salud, Universidad Icesi, Cali, Colombia; ^3^Genetics Service, Fundación Valle del Lili, Cali, Colombia; ^4^Department of Radiology, Fundación Valle del Lili, Cali, Colombia; ^5^Centro de Investigaciones en Anomalías Congénitas y Enfermedades Raras (CIACER), Universidad Icesi, Cali, Colombia

**Keywords:** vascular Ehlers–Danlos syndrome, *COL3A1*, intracranial aneurysm, rare disease, Colombia, case report

## Abstract

**Introduction:**

To date, approximately 600 unique pathogenic variants have been reported in *COL3A1* associated with vascular Ehlers–Danlos syndrome (vEDS). The objective of this study was to describe a patient with a novel variant in *COL3A1* associated with vEDS.

**Case report:**

We describe the clinical history and thorough phenotyping of a patient with brain aneurysms and identified a novel pathogenic variant in *COL3A1*. This male patient reported transient focal neurologic symptoms. Physical examination showed abnormal atrophic scarring, horizontal stretch marks under the arms, and an acrogeric appearance of the skin of the hands and feet. Brain imaging revealed extensive dilation of both internal carotids and the vertebrobasilar system. Molecular analysis identified a variant in *COL3A1* (NM_000090.4):c.3058G>T p.(Gly1020Cys), which was classified as likely pathogenic. Currently, the patient has never had an event concerning dissection/rupture of tissues that could be affected in this condition.

**Conclusion:**

This report demonstrates that exhaustive evaluation with clinical and genetic approaches should be considered in patients with vascular abnormalities. vEDS has a variable clinical presentation and often goes unrecognized, even though it is related to life-threatening complications and a shortened life expectancy. Diagnosis confirmed by genetic testing is crucial to determining appropriate surveillance, prevention, treatment, and genetic counseling.

## 1 Introduction

Vascular Ehlers–Danlos syndrome (vEDS) or EDS type IV is a rare connective tissue disorder of the heterogeneous group of EDS, characterized by intracranial and extracranial vascular abnormalities (1). vEDS is the least common form of EDS, with an estimated prevalence of 1/150,000. The major neurological complications include carotid cavernous fistulas, cervical arterial dissections, and intracranial aneurysms (2, 3).

**Figure 1:**
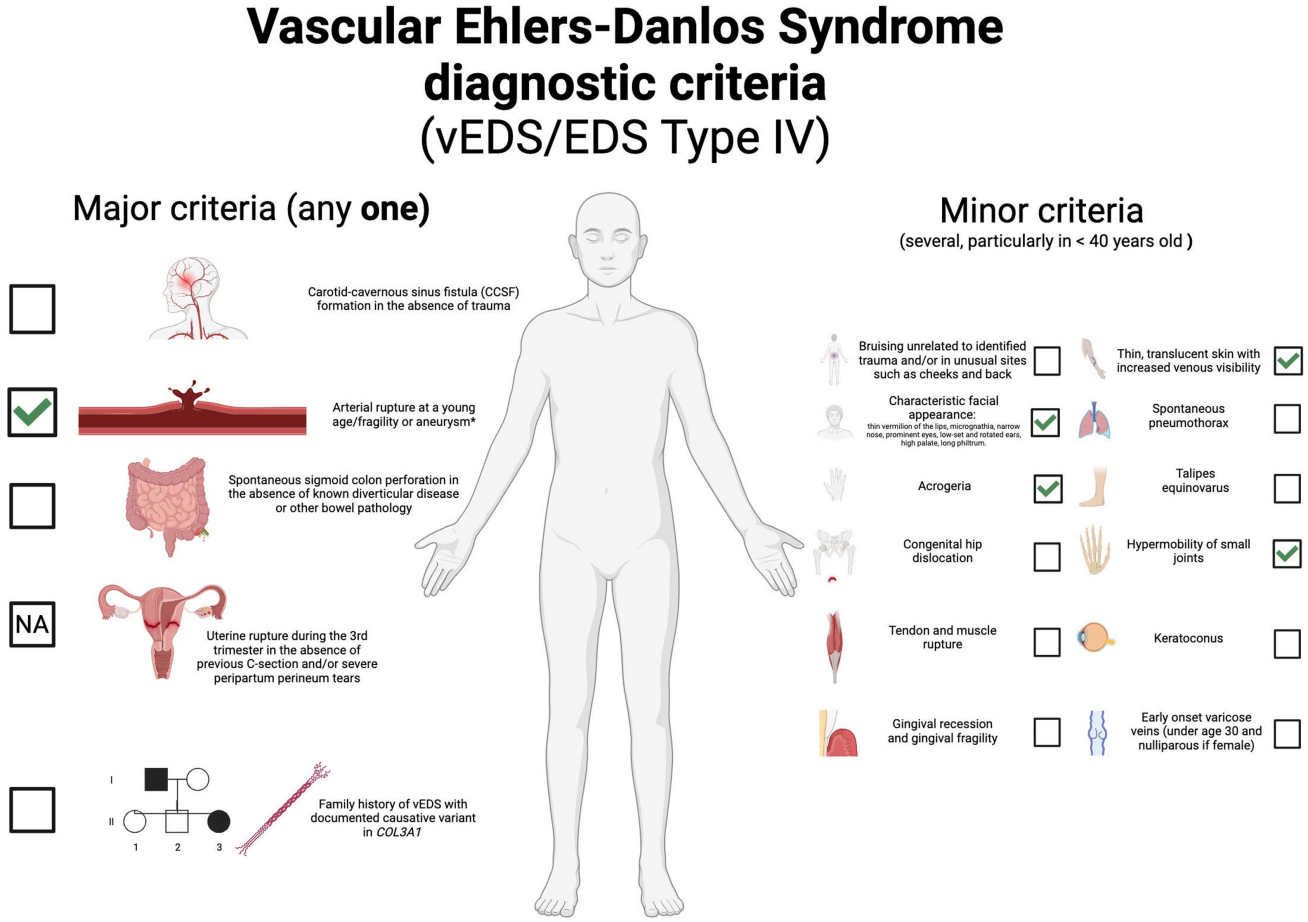
Vascular Ehlers-Danlos Syndrome diagnostic criteria. According with “The 2017 international classification of the Ehlers-Danlos syndromes”. Minimal criteria suggestive for vEDS: A family history of the disorder, arterial rupture or dissection in individuals less than 40 years of age, unexplained sigmoid colon rupture, or spontaneous pneumothorax in the presence of other features consistent with vEDS should all lead to diagnostic studies to determine if the individual has vEDS. Testing for vEDS should also be considered in the presence of a combination of the other “minor” clinical features. (✔): positive or presented in our case. (NA): not applicable. (*): some authors reported fragility or aneurysm in tissues.

Diagnosing vEDS is challenging due to its uncommon occurrence and diverse clinical manifestations. It relies on clinical observations initially and is later confirmed through molecular testing. The clinical identification of vEDS can be challenging as it shares similarities with other connective tissue disorders and arterial pathologies. For individuals with a history of early-age arterial rupture or dissection, intestinal rupture, or idiopathic pneumothorax, suspicion of vEDS should arise if they exhibit minor physical traits such as a thin, see-through skin with prominent veins and increased flexibility in small joints. Figure 1 shows vEDS diagnostic criteria (1). Diagnosis of vEDS is confirmed with a positive genetic test, that is, the identification of a pathogenic variant in *COL3A1*, which encodes the pro-α 1 chain of type III procollagen. Currently, more than 600 unique pathogenic variants in *COL3A1* have been identified (4, 5).

The most frequent variants are heterozygous missense substitutions affecting glycine residues, but in some cases, vEDS can also stem from modifications in the splicing sites of genetic segments responsible for encoding a sequence forming a triple helix. Rarer variations such as frameshift mutations, nonsense mutations, or large deletions can also be found (5). An accurate diagnosis, coupled with suitable clinical care and ongoing monitoring, enhances the survival rates of individuals with vEDS. We report a case of vEDS associated with a novel missense variant in exon 42 of the *COL3A1* gene in a patient with isolated cerebrovascular involvement.

## 2 Case description

**Figure 2:**
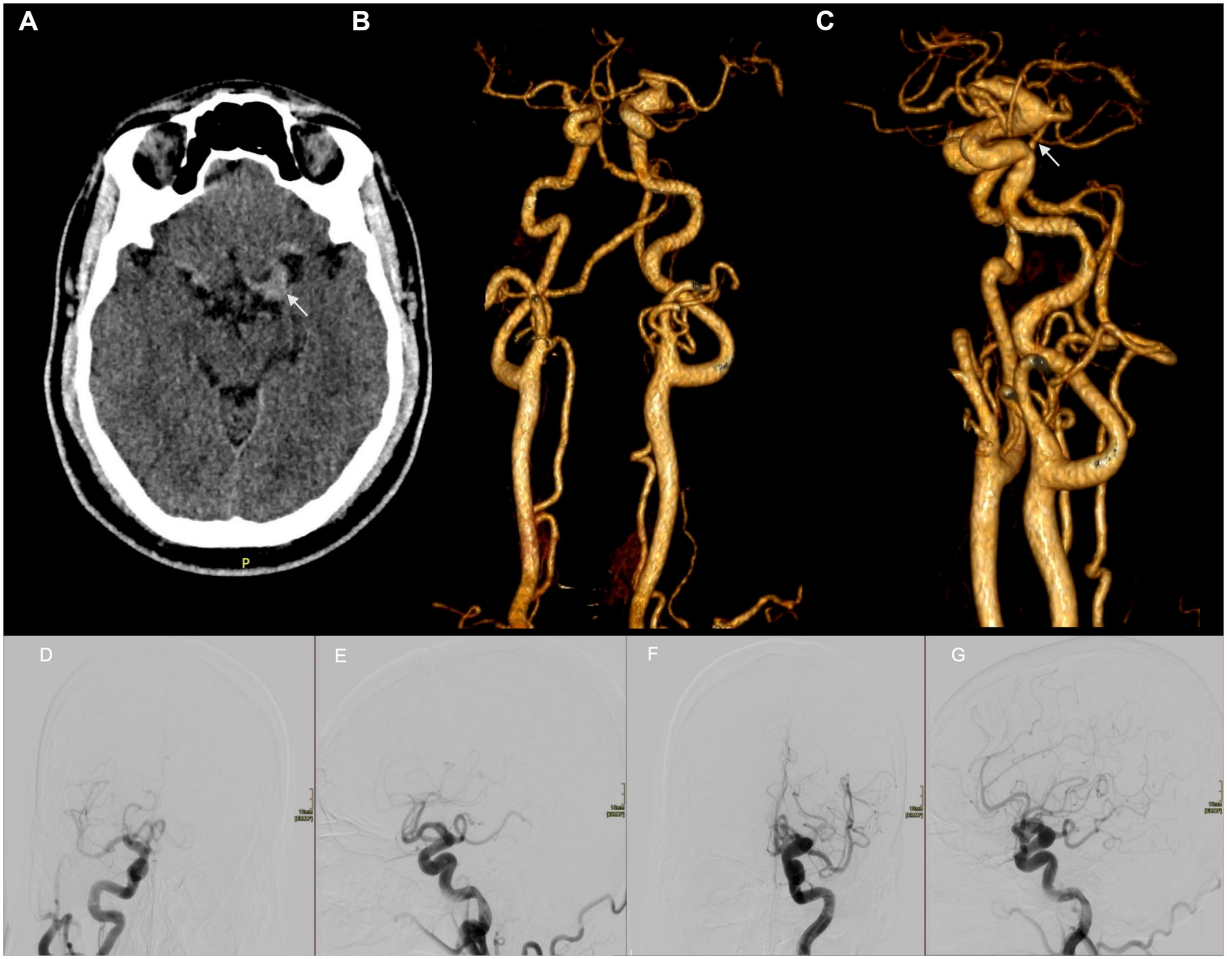
**(A)** An axial non-contrast CT scan shows a fusiform aneurysm of 10 mm diameter of the M1 segment of the left middle cerebral artery with increased density. **(B)** A three-dimensional (3D) reconstruction of CT angiography of the cerebral arteries shows a fusiform aneurysm involving the ophthalmic and posterior communicating segments of the left internal carotid artery. The course of both internal carotid arteries and the vertebrobasilar system is tortuous. **(C)** A lateral view of the 3D reconstruction of CT angiography of the cerebral arteries. Cerebral angiography. AP **(D,F)** and lateral projections **(E,G)** of internal carotids showing fusiform dilation and tortuosity of both internal carotid arteries from the petrous segment to the terminal segment. No saccular aneurysms are seen. **(D,E)** Right carotid. **(F,G)** Left carotid.

We present the case of a 38-year-old man with resistant hypertension who presented to the emergency department (ED) after transient right-arm monoparesis that lasted 25–30 min. On admission, his blood pressure was 180/115 mmHg, and the neurological examination was normal. Head computed tomography (CT) was ordered to exclude the possibility of a stroke; brain parenchyma showed no abnormalities, but a fusiform dilation of the M1 segment of the left middle cerebral artery with increased density was observed (Figure 2A). CT angiography of the cerebral arteries showed tortuosity of the internal carotid arteries (Figures 2B,C). Cerebral angiography confirmed an extensive dilation of both internal carotids and the vertebrobasilar system with no lesions requiring endovascular treatment and suggestive of a systemic etiology (Figures 2D–G). Abdominal CT angiography showed no vascular abnormalities. Transesophageal echocardiogram showed no alterations, and all metabolic, autoimmune, and infectious tests were normal. The patient was discharged with a diagnosis of a transient ischemic attack and instructions for secondary prevention and outpatient follow-up.

**Figure 3:**
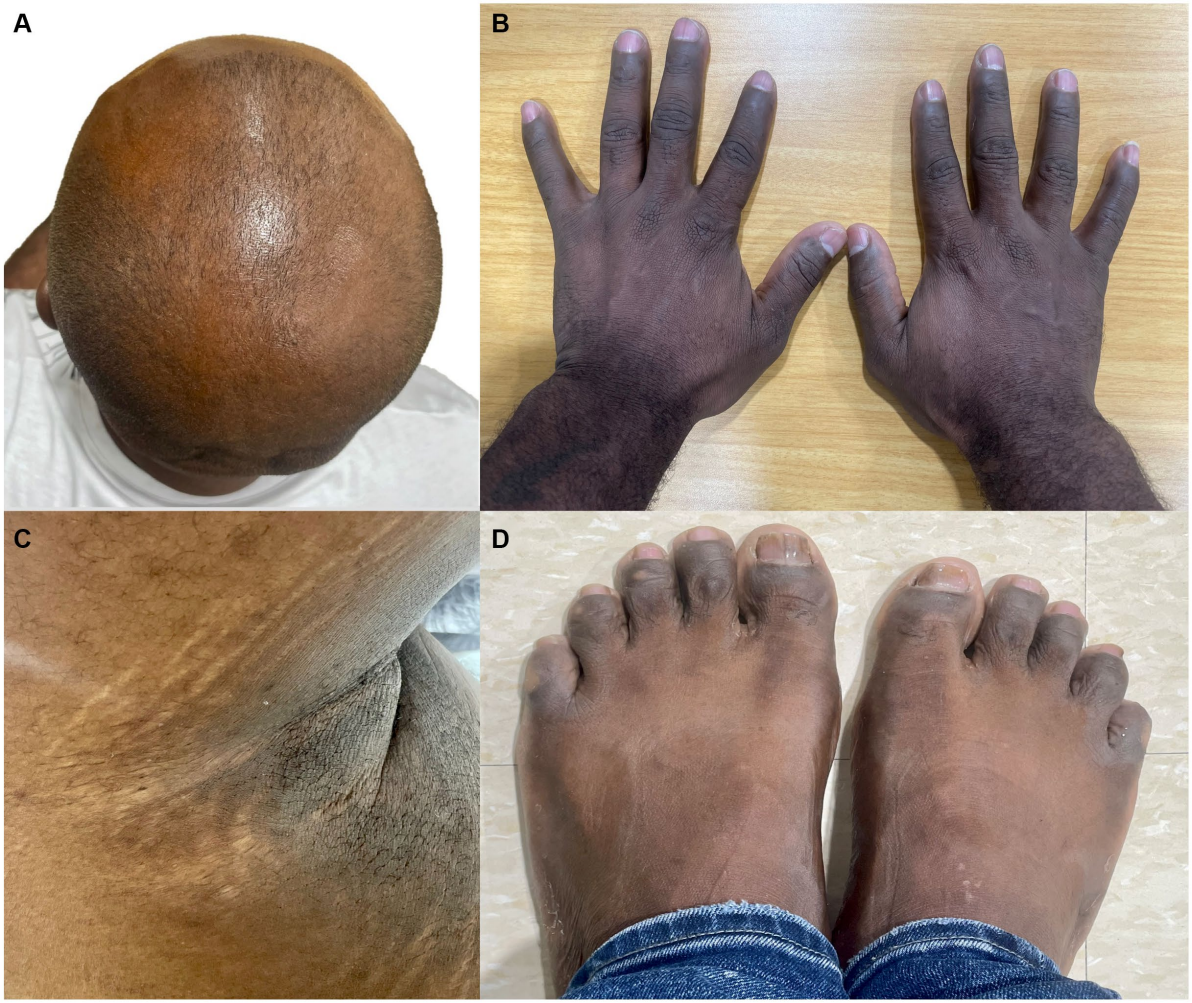
Patient had alopecia of the scalp **(A)**, horizontal stretch marks under the arms **(C)**, and acrogeric appearance of the skin of the hands and feet **(B,D)**.

Four months later, he presented again to the ED for a syncope due to a hypertensive crisis without focal neurologic symptoms. The images from the brain CT and CT angiography showed no discrepancies compared to the previous images. A Doppler ultrasound of renal arteries reported no stenosis. Oral antihypertensives were adjusted, and he was discharged. Given the diffuse involvement of the cerebral vasculature, the patient was referred to medical genetics. The patient had no relevant family health history or familial hypertension history. The parents of the proband were not available; his father died in a traffic accident at the age of 61, and his mother had been living in another city for several years. Physical examination revealed thin lips, abnormal atrophic scarring, thin skin with venous visibility in some areas, alopecia of the scalp, horizontal stretch marks under the arms, and acrogeric appearance of the skin of the hands and feet (Figure 3). The Beighton score was 4 points. The rest of the examination was unremarkable.

**Figure 4:**
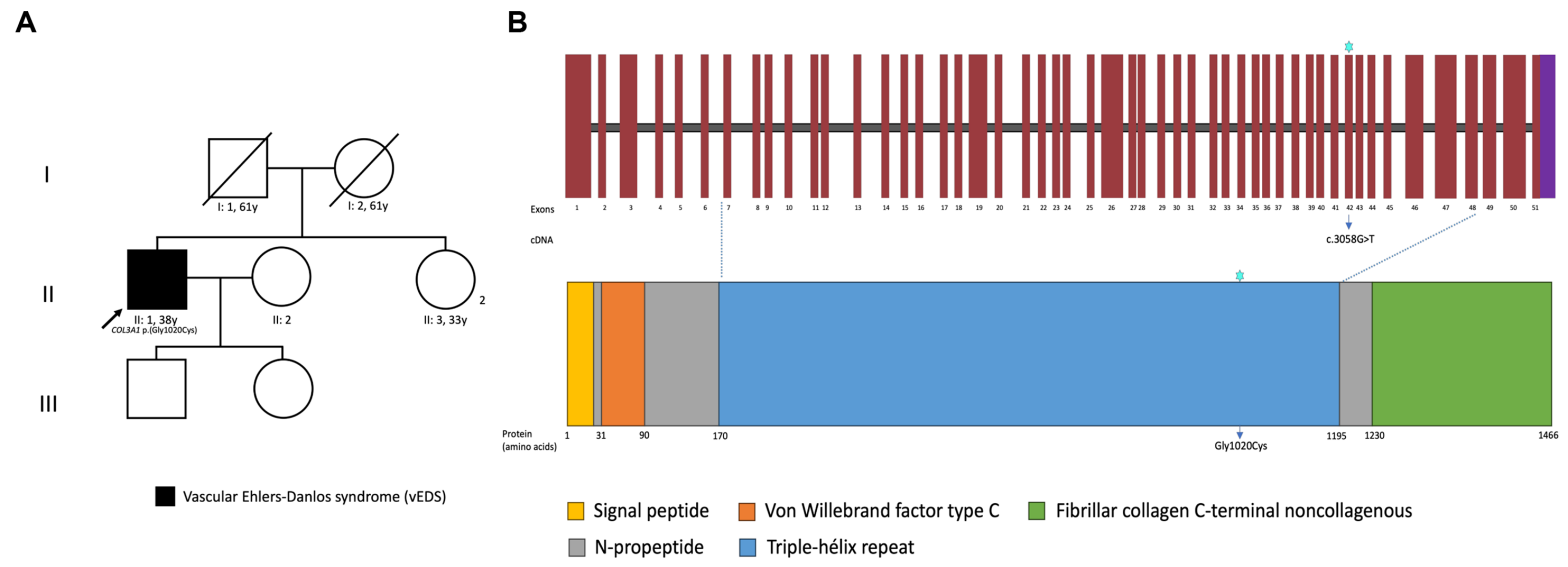
Pedigree of the patient and family **(A)**, schema of the *COL3A1* protein structure, its domains, and localization of the identified variant **(B)**.

Proband-only exome sequencing was performed and reported a heterozygous variant in *COL3A1* (NM_000090.4):c.3058G>T p.(Gly1020Cys) at genomic coordinates GRCh38 chr2:189006224, which was later confirmed by Sanger sequencing. This variant has not been annotated in the gnomAD, the ExAC, 1,000 Genomes, ClinVar, or HGMD databases. According to the American College of Medical Genetics and the Association for Molecular Pathology (ACMG/AMP) guidelines, we classified this variant as likely pathogenic, and a diagnosis of vEDS was made (1, 6). Figure 4 shows the patient’s pedigree, a schema of the *COL3A1* gene and protein, and the site of the variant.

## 3 Methods

Informed consent from the patient was obtained, reviewed, and approved by the Ethics Committee of Fundación Valle del Lili, Colombia (human study protocol #487-2023). The study was conducted in accordance with the Declaration of Helsinki.

A peripheral blood sample was obtained from the proband, and DNA was extracted using a Qiagen QIAamp® DNA Mini Kit. DNA was sequenced using the GeneSGKits® protocol for next-generation sequencing (NGS). Exome sequencing was performed. Target regions were captured using the SureSelectXT Human All Exon V6 (Agilent Technologies), and sequencing was performed on an Illumina HiSeq® instrument. The sequencing reads were aligned to the human reference genome (GRCh38) using the Burrows–Wheeler Aligner.

The Genome Analysis Toolkit (GATK) was used to perform variant calling (VCF_files). Variants were filtered using GeneSystems® analysis software according to whether it was a gene with the Online Mendelian Inheritance in Man (OMIM) phenotype. The analyzed regions include the coding exons and adjacent intronic regions (±10 bp) of the captured genes. Only variants with allele frequency < 1% were considered. The Integrative Genomics Viewer was used for visual exploration of the clinically relevant variants. Following this process, we were able to get a list of 32 potential variations. Additionally, a file was prepared containing genes that are related or potentially related to possible major phenotypes based on Human Phenotype Ontology (HPO) terms. This file served as a selected list of candidate genes for variant filtering and included the following list of terms: “Hypertension HP:0000822”; “hypertensive crisis HP:0100735”; “cerebral aneurysm HP:0004944”; “carotid artery aneurysm HP:0012163”; “aneurysm HP:0002617; “transient ischemic attack HP:0002326″; “joint hypermobility HP:0001382″; “metacarpophalangeal joint hyperextensibility HP:0006099″; and “atrophic scars HP:0001075″. Variants with a high pathogenicity score were selectively retained from the output file, and only the *COL3A* variant was prioritized after the phenotype HPO terms analysis. The read count was ≥20x, and a variant reads/total reads ratio ≥ 0.2. ACMG/AMP criteria were used for variant classification (6). Finally, according to the similarity between clinical features in the patient and the gene–phenotype relationship, Sanger sequencing for the *COL3A1* variant was performed. A 3730xl DNA Analyzer (Applied Biosystems) was used, and the sequence of the primers was as follows: forward 5′-GGTGCAATCATGGCTCACTT-3′ and reverse 5′-CCTTTGTAAGTCAGACAGGTTG-3′.

## 4 Discussion

The clinical diagnosis of vEDS is often challenging due to its overlap with other connective tissue disorders and arteriopathies. Typically, vEDS is suspected in cases involving severe manifestations or premature death occurring before the fifth decade, characterized by spontaneous ruptures of the arteries, uterus, or bowel, although it has also referred to the fragility of tissues, such as arterial aneurysms that precede dissections or ruptures (7). However, the individual we present had a distinct clinical scenario where he exhibited only arterial aneurysms, thin vermillion of the lips, hypermobility of small joints (Beighton score 4 for fifth fingers and thumbs), thin skin, and acrogeria, serving as major and minor diagnostic criteria for vEDS, respectively (Figure 1) (1, 7). Our patient exhibits syndrome features that enable the establishment of a diagnosis through a multidisciplinary approach, including genetic consultation and testing to identify a novel variant in *COL3A1*, expanding the spectrum of disease-causing variants. The *COL3A1* gene (MIM: *120180) is located on chromosome 2q32.2 and spans 44 kb, comprising 51 exons. It encodes the pro-α (III) chain of type III collagen, a protein characterized by 343 repetitions of glycine-X-Y (Gly-Xaa-Yaa) in each of the three polypeptide chains. Type III collagen is primarily found in connective tissues such as skin, lungs, uterus, intestine, and the vascular system, often in conjunction with type I collagen (8). More than 600 unique pathogenic variants have been reported in *COL3A1* associated with vEDS (9). They have been classified as glycine substitutions, splice-site, frameshift, nonsense, large deletion variants, and non-glycine missense variants, including those within or outside the triple helix or in-frame indels in the N-or C-terminal part of the protein (5).

In our patient, a missense variant in exon 42 of the *COL3A1* gene was detected, c.3058G>T, resulting in a Gly-to-Cys substitution at position 1,020 of the protein. Multiple *in silico* scores support the interpretation that this substitution is deleterious (MetaRNN = 0.997, CADD = 32, PolyPhen-2 = 1, SIFT = 0, REVEL = 0.993; PP3). The variant is missense in a gene with a low rate of benign missense mutations, where this type of variant is a common mechanism of disease (PP2, moderate). The variant is in a mutational region of interest and well-established functional domain with missense variants reported (PM1, moderate). An alternative variant (p.Gly1020Asp) is classified as pathogenic by LOVD (PM5, moderate); and the variant is absent from gnomAD (PM2, supporting). Taken together, these data result in a likely pathogenic classification (PP3, PP2_Moderate, PM1_Moderate, PM5_Moderate, and PM2_Supporting) according to the ACMG/AMP guidelines (6), as it has not been reported in the literature. However, the c.3059G>A p.(Gly1020Asp) variant has been described at the same exon and position of the protein (4), though they appear to be unrelated as cysteine is a neutral amino acid, whereas aspartic acid (Asp) is acidic.

In previous studies, a stability-related bias was considered in glycine-substituted residues of the collagen triple helix (Gly-Xaa-Yaa) in inherited connective tissue disorders. It was observed that certain amino acids, including Cys, Ser, and Asp., were involved in destabilizing mutations more frequently than expected (10). This trend has been observed in *COL3A1* substitutions, where alanine (Ala) and Ser replace glycine less frequently than expected, and valine (Val), glutamic acid (Glu), and Asp replace it more often than expected (11). Interestingly, the substitution of Val or asparagine (Asn) for glycine has been associated with a poorer prognosis compared to a substitution of Ser (12).

Given the extreme tissue fragility associated with vEDS, severe cases of the syndrome are not unexpected and have been reported to manifest in patients between the ages of 30 and 40 years, displaying previously described clinical features. In cases involving arterial ruptures, these clinical features are commonly preceded by aneurysms, as seen in our case, with rupture sites primarily occurring in the chest and abdomen (66%), followed by the head and neck (17%), and extremities (17%) (13). Additionally, it is important to highlight the possibility of non-syndromic arteriopathy presentation without extra-arterial characteristics suggestive of vEDS, with evidence of phenotypic variability related to descriptions of both glycine-substituted and haploinsufficiency variants in *COL3A1* (14).

Following the diagnosis of the patient, an integral multidisciplinary approach and surveillance were conducted to investigate the phenotypic findings described in previous reports. This investigation involved mainly non-invasive imaging to identify aneurysms, dissections, or vascular ruptures, as well as blood pressure monitoring. Effective interventions were employed to reduce the risk of arterial dissection or rupture and prolong life, such as management of blood pressure, and recommendations of circumstances to avoid such as trauma, arteriography, routine colonoscopy, and elective surgery (2, 15). Other recommendations for patients who have vEDS include regular, low-intensity exercise and annual imaging of the thoracic and abdominal aorta, cervical vessels, including the circle of Willis, and distally to the pelvis/upper legs (16). The identification of evolving changes must be assessed with specific imaging at shorter intervals. During a follow-up examination after 1 year, he did not have adverse vascular events and family studies are pending.

Evidence suggests that, having an early and correct diagnosis made, appropriate clinical management and long-term follow-up improve survival for patients who have vEDS (11, 13, 14, 16). This has been possible in part because the assessments by specialists, including geneticists, together with studies such as exome sequencing, are part of the services and technologies financed or covered by Colombia’s health benefits plan guaranteed to all persons affiliated with the Social Security Health System. Assessing and counseling the family members, including seemingly unaffected older and younger at-risk relatives, are suitable to promptly identify those who could benefit from early surveillance, promote treatment awareness for possible complications, and adopt risk-reducing behaviors such as limiting high-risk physical activities for family planning purposes.

## 5 Conclusion

We emphasize the role of a multidisciplinary workup in effectively managing vEDS, where an early diagnosis allows the implementation of appropriate medical management that can significantly enhance the survival and quality of life for individuals affected by this condition. vEDS should be suspected in young patients with transient focal neurological symptoms and aneurysmal dilatations of cerebral vasculature because clinical suspicion allows early genetic testing for connective tissue disorders.

## Data availability statement

The datasets for this article are not publicly available due to concerns regarding participant/patient anonymity. Requests to access the datasets should be directed to the corresponding author.

## Ethics statement

The studies involving humans were approved by IRB Biomedical Research Ethics Committee of the Fundacion Valle del Lili. The studies were conducted in accordance with the local legislation and institutional requirements. The participants provided their written informed consent to participate in this study and for the publication of any potentially identifiable images or data included in this article.

## Author contributions

VV-C: Writing – review & editing, Writing – original draft, Methodology, Formal analysis, Conceptualization. SS-D: Writing – review & editing, Writing – original draft, Methodology, Formal analysis, Conceptualization. VQ-P: Writing – review & editing, Data curation. EF: Writing – review & editing, Data curation. JN-C: Writing – review & editing, Supervision, Methodology, Formal analysis. HP: Writing – review & editing, Supervision, Methodology, Formal analysis. JF-C: Writing – review & editing, Supervision, Conceptualization.
